# Editorial: Seed dormancy, germination, and pre-harvest sprouting, volume II

**DOI:** 10.3389/fpls.2024.1399510

**Published:** 2024-03-26

**Authors:** Yong Jia, Jose Maria Barrero, Jirui Wang, Michael James Considine, Shingo Nakamura, Chengdao Li

**Affiliations:** ^1^ Western Crop Genetics Alliance/State Agricultural Research Centre, School of Agriculture, Murdoch University, Murdoch, WA, Australia; ^2^ Agriculture and Food, Black Mountain Science and Innovation Park, Commonwealth Scientifc and Industrial Research Organisation (CSIRO), Canberra, ACT, Australia; ^3^ Triticeae Research Institute, Sichuan Agricultural University, Wenjiang, Chengdu, Sichuan, China; ^4^ The UWA Institute of Agriculture, School of Molecular Sciences, University of Western Australia (UWA), Crawley, WA, Australia; ^5^ Division of Crop Design Research, Institute of Crop Science, NARO, Tsukuba, Japan

**Keywords:** seed dormancy, pre-harvest sprouting, late maturity alpha-amylase, crop, molecular mechanism, germination

Many plant species have acquired seed dormancy during evolution to maximise their fitness and survival. This adaptive trait has many evolutionary benefits such as allowing seed dispersal, preventing germination in unfavourable conditions, and improving survival rate across multiple conditions (Koornneef et al., 2002; Venable, 2007). However, seed dormancy has generally been lost or weakened during crop domestication due to artificial selection for rapid and uniform germination (Hammer, 1984). And this loss of seed dormancy makes modern cultivated crops particularly susceptible to a grain defect called pre-harvest sprouting (PHS).

PHS describes the phenomenon of seed germination in the mother plants, before harvest, while the seeds have not been released yet (Mares and Mrva, 2014). This is a very important problem in cereals crops, as grains affected by PHS are characterised by increased levels of alpha-amylase activity and low falling numbers (FN), which lead to poor processing quality and immediate grain downgrading (Mares and Mrva, 2014). In agriculture industry, PHS has been noted as a severe grain defect globally, causing annual economic loss over a billion of dollars worldwide (Black et al., 2006). The occurrence of PHS generally requires a humid condition and a reduced seed dormancy during or after the grain maturity stage (Mares and Mrva, 2014). This process has a complex genetic, biochemical, and molecular basis, and closely interacts with environmental factors such as rainfalls and temperature (Mares and Mrva, 2014; Tai et al., 2021). Abscisic acid (ABA) and gibberellin acid (GA) are two critical plant hormones inducing seed dormancy and seed germination, respectively, thus their metabolic and signalling pathways have been the main targets of most of the studies dedicated to understand and to solve the PHS issue (Finkelstein et al., 2008). In the context of breeding for PHS-resistant genotypes, the ultimate solution to PHS damage must be a delicate manipulation of seed dormancy without sacrificing a germination rate and plant establishment or other agronomic traits such as yield. Over the last few decades, PHS has continuously attracted enormous attention from crop farmers, breeders, and plant scientists.

This Research Topic “*Seed dormancy, germination, and pre-harvest sprouting, volume II*” is part of the “Seed dormancy, germination, and pre-harvest sprouting” series hosted by *Frontiers in Plant Science*. The first series (Nonogaki et al., 2018) covered the full papers and abstracts from the 13^th^ International Symposium on Pre-harvest Sprouting in Cereals (ISPSC, Perth, Australia 2016) and other dedicated papers at the time. Since then, the 14^th^ and 15^th^ ISPCS have been held in 2019 (Chengdu, China) and 2023 (Tsukuba, Japan), respectively, both attracting enormous interests from relevant researchers from around the world. The last ten years have seen significant advancements in new genomic resources, genotyping and sequencing, phenotyping, and multi-omics that are allowing the breeding of PHS-tolerant varieties. We have witnessed many novel and exciting findings in our understanding of the genetic and molecular basis associated with seed dormancy and PHS.

In cereal crops such as wheat and barley, the problem of PHS can be confounded with others known as late maturity alpha-amylase (LMA) or vivipary. These traits cause elevated alpha-amylase in the ripened grain, leading to low FN and grain quality downgrading (Mares and Mrva, 2014; Peery et al.). As mentioned, PHS generally results from insufficient grain dormancy at maturity in combination with high moisture. It is intrinsically associated to the germination process, when the alpha-amylase is induced by GA when germination has started in matured grains. In contrast, LMA or vivipary refers the induction of alpha-amylase at the soft dough stage when grains are still unmature. In view of their field observation that wheat grain can germinate precociously during grain development, Peery et al. examined whether LMA and vivipary are related. They found that visible germination of immature grain in the soft to hard dough stages (Zadok’s stages 83-87) could be strongly induced under cool and humid conditions used for the induction of LMA, which results in similar pattern of alpha-amylase expression with LMA but varies from that for vivipary. However, they also noted that wheat varieties susceptible to premature seed germination (vivipary) were not always associated with LMA susceptibility, implying distinct molecular mechanisms.

One effective approach to dissect the elusive role of alpha-amylase in seed dormancy and seed germination is through transgenic over-expression or knock-down of relevant genes. In the wheat genome, there are four isoforms of alpha-amylase (TaAMY1, TaAMY2, TaAMY3, and TaAMY4) (Ral et al., 2016). Earlier studies have already indicated that these different isoforms displayed different substrate specificity and expression patterns, thus they may have different impact on seed germination (Cockburn et al., 2015). In view of this, Zhang et al. performed overexpression of TaAMY1 in wheat and examined its impact on starch structure and seed dormancy. Using an endosperm-specific promoter, they developed an approach to express TaAMY1 specifically in wheat grain, despite some leaked expression in the stem and leaf tissues with minimal impact. They found that the accumulation of TaAMY1 caused a higher degree of damaged starch in the late stage of grain development but barely affected the starch visco-properties, which could be fully restored with an alpha-amylase inhibitor. In contrast, previous overexpression of TaAMY2 and TaAMY3 displayed much lower recovery rates, implying different impacts for the different alpha-amylase isoforms. Finally, they showed that the elevated TaAMY1 in wheat grain could indeed reduce seed dormancy and enhanced ABA resistance, both of which were associated with the accumulation of soluble sugar (alpha-gluco-oligosaccharide and sucrose).

The regulation of seed dormancy and seed germination is critical for the effective prevention of PHS. After grain maturity, dormancy can be lost through dry after-ripening or cold stratification when imbibing under cool temperature. As mentioned before, this process is controlled predominantly by two antagonistic plant hormones: the dormancy-promoting ABA and the germination-promoting GA. In *Arabidopsis*, seed dormancy has been shown to be associated with higher level of ABA at the late timepoint of imbibition. However, the corresponding profile for GA, which occurs in much lower level than ABA and is often difficult to detect, remain to be characterised. Employing an optimised sensitive GA detection method, Nelson et al. measured GA levels in dry seeds and in imbibing seeds before germination and found clear evidence that the increased germination capacity and dormancy loss happening during after-ripening was associated with increased levels of GA_4_. In addition, they also observed that ABA contents only decreased later in imbibition, just before germination. Based on these observations, they proposed that GA may act first to stimulate seed germination before the decline of ABA, which appears to act as the final checkpoint preventing germination before other processes essential for seed survival are completed, such as DNA repair and activation of respiration. Moreover, their data also indicated that *Arabidopsis* gene *GID1b* is positive regulator of germination in the *sly1-2* mutant but a negative regulator in the wild type.

To date, most studies on seed dormancy and PHS prevention have focused on major cereal crops such as wheat, barley, rice, maize, and model species such as *Arabidopsis*. However, the PHS issue in some economically less important crops also deserves attention. For example, quinoa (*Chenopodium quinoa* Willd.) has recently emerged as a popular staple food due to its nutritional properties (Murphy et al., 2016). This crop has also been noted for its exceptional stress tolerance and ability to grow in marginal soils and harsh climates, thus has great significance for global food security in the context of climate change (Murphy et al., 2016; Nadali et al., 2021). In a ground-breaking work, McGinty et al. developed a dormancy assay and screened a large collection of quinoa varieties. They also investigated the impact on seed dormancy of seed traits such as seed coat colour, seed coat thickness, seed shape, protein content, seed moisture and the sensitivity to exogenous application of GA and ABA. They found that commercial quinoa varieties range from no dormancy to strong dormancy, and seed coat thickness and eccentricity are particularly important traits affecting seed dormancy levels. Additionall, Zeng et al., (2024) characterised the dynamic profiles of eight endogenous hormones during quinoa seed germination using the HPLC-ESI-MS/MS and ELISA method, complemented with transcriptome network analyses using RNA-seq. They found that quinoa shared some common metabolic patterns with other crops during seed germination, but also exhibited a few quinoa-specific hormone changes that will require further attention. Both studies have significantly improved our understanding of seed dormancy in quinoa and will facilitate future development of PHS prevention strategies and the breeding of PHS-tolerant quinoa varieties.

In addition to quinoa, mungbean (*Vigna radiata* L.) is another relatively “small” crop for which PHS has been recognised as a prominent limiting factor. Major quantitative trait loci for seed dormancy in mungbean have been reported in several previous studies (Humphry et al., 2005; Isemura et al., 2012). However, the underlying candidate genes remain elusive. Laosatit et al. employed a F2 population derived from a dormant wild mungbean “ACC41” and a non-dormant cultivated mungbean “Kamphaeng Saen 2” and performed fine mapping of the *Sdwa5.1.1C* locus. They identified VrKNAT7-1, encoding a transcription factor KNOTTED ARABIDOPSIS THALIANA7 (KNAT7), a class II KNOTTED1-LIKE HOMEOBOX (KNOX II) protein, as the underlying candidate gene, which was found with higher expression in the seed of ACC41 than that in “Kamphaeng Saen 2”, most prominently in the seed coat. In addition, they noted that the seeds of wild mungbean exhibited a unique palisade cuticle layer, which is absent in cultivated mungbean, highlighting the potential impact of seed anatomy on dormancy.

We would like to acknowledge the above authors for their dedicated interests and valuable contribution to this Research Topic. We believe “Seed Dormancy, Germination, and Pre-Harvest Sprouting – Volume II” serves as a timely follow-up of the first series back in 2018 (Nonogaki et al., 2018) and have captured some important new findings and progresses relevant to seed dormancy and PHS-prevention in different plants. We noted several key features from this collection of publications: 1) Novel insights into the molecular mechanisms of PHS, LMA, and seed germination in model species are continuously being made. For example, the work by Peery et al. showed that LMA and immature grain germination are distinct processes despite a similar alpha-amylase expression pattern. Nelson et al. proposed an interesting perspective that ABA acts the final checkpoint preventing germination in the late stage of imbibition. 2) Improved analytical approaches and genomic resources have assisted new research in this area, such as the optimised hormone detection method used by Nelson et al., the transcriptome sequencing by Zeng et al., 2024 and Nelson et al., and the genetic map and genome assembly for mungbean used by Laosatit et al.. 3) genome association analyses and transgenic overexpression prove as effective approaches to examine the molecular processes underlying PHS. 4) Whilst major cereal crops remain dominant, particularly wheat and rice, researchers working in other non-cereal crops are paying increasing attention to the PHS topic. These crops are not only quinoa and mungbean as reported in this Research Topic, but also include other crops such as buckwheat (Suzuki et al., 2021) and cucumber (Cao et al., 2021). 5) In addition to the molecular mechanisms, physical morphology traits such as seed coat thickness, grain colour, and seed shape prove to have important impact on seed dormancy and may be targeted for effective PHS prevention.

Finally, a few other important directions in PHS-related research not covered in this Research Topic but should be pointed out include: CRISPR/Cas9 mediated genome editing, the use of multi-omics (genome, transcriptome, proteome, metabolome, and epigenome) approach, interaction of environment with PHS, and discovery of new PHS-tolerant genetic materials and their use in breeding. Despite the enormous progress made to date, we predict that the PHS and LMA will continue to be a serious problem for many crops, and that under the increasingly volatile climate conditions, new knowledge gaps and challenges will appear and persist. We expect these challenges should be addressed by concerted efforts from plant researchers, breeders, farmers, and the relevant industry players.

## Author contributions

YJ: Writing – original draft. JB: Writing – review & editing. JW: Writing – review & editing. MC: Writing – review & editing. SN: Writing – review & editing. CL: Writing – review & editing.
